# The Impact of Technical Innovations and Donor-Site Mesh Repair on Autologous Abdominal-Based Breast Reconstruction—A Retrospective Analysis

**DOI:** 10.3390/jcm13082165

**Published:** 2024-04-09

**Authors:** Theresa Promny, Paula Huberth, Wibke Müller-Seubert, Dominik Promny, Aijia Cai, Raymund E. Horch, Andreas Arkudas

**Affiliations:** Department of Plastic and Hand Surgery and Laboratory for Tissue Engineering and Regenerative Medicine, University Hospital Erlangen, Friedrich-Alexander University Erlangen-Nürnberg (FAU), 91054 Erlangen, Germany; paula.huberth@uk-erlangen.de (P.H.); wibke.mueller-seubert@uk-erlangen.de (W.M.-S.); dominik.promny@uk-erlangen.de (D.P.); aijia.cai@uk-erlangen.de (A.C.); raymund.horch@uk-erlangen.de (R.E.H.); andreas.arkudas@uk-erlangen.de (A.A.)

**Keywords:** DIEP, MS-TRAM, perforator flaps, indocyanine green angiography, computed tomography angiography, coupler anastomoses, synthetic mesh

## Abstract

**Background**: The aim of this study was to examine the potential benefit that may be achieved through the introduction of technical innovations and the incorporation of mesh for fascial donor site closure in uni- and bilateral autologous breast reconstruction with abdominal tissue. **Methods**: A retrospective single-center review of all breast reconstructions with a DIEP or MS-TRAM flap between January 2004 and December 2019 was performed. Donor and recipient site complications and operation times were evaluated before and after the implementation of coupler anastomoses, preoperative computed tomography angiography (CTA), indocyanine green (ICG) angiography, and the inclusion of mesh in donor site repair. **Results**: A total of 396 patients were included, accounting for 447 flaps. Operation time was significantly shorter in unilateral reconstructions after the implementation of CTA (*p* < 0.0001). ICG angiography significantly reduced the rates of partial flap loss (*p* = 0.02) and wound healing disorders (*p* = 0.02). For unilateral reconstructions, abdominal bulging or hernia was observed more often in MS1-TRAM flaps without synthetic mesh repair (*p* = 0.001), whereas conservatively treated seroma developed more frequently after mesh implantation (*p* = 0.03). **Conclusions**: Recent technological advancements developed over the past few decades have made a substantial impact on decreasing surgical duration and enhancing procedure safety.

## 1. Introduction

Autologous breast tissue reconstruction with an abdominal free flap based on the inferior epigastric vessels, the free transverse rectus abdominis myocutaneous (TRAM) flap, was first described by Holmström in 1979 [1]. In the following years, the muscle-sparing TRAM (MS-TRAM) was developed to reduce abdominal wall morbidity [2]. In 1994, Allen and Treece described the reconstruction of a breast by transferring skin and fat from the lower abdomen based on the deep inferior epigastric vessels and without the sacrifice of the rectus abdominis muscle—the deep inferior epigastric perforator (DIEP) flap [3]. Since then, not only refinements of surgical techniques but also technical devices have been developed to optimize success rates and the appearance of the reconstructed breast as well as to reduce donor site morbidity. One reason for flap revision or even flap loss can be venous congestion due to problems with microvascular anastomosis. Mechanical venous coupler devices as an alternative to hand-sewn venous anastomosis have gained significance in free flap surgery and have been used routinely by many reconstructive microsurgeons within recent decades [4,5,6,7]. Further complications might be partial or complete postoperative flap necrosis. Therefore, laser-assisted indocyanine green (ICG) fluorescence angiography has been established in free tissue transfer to visualize blood flow in the tissue of interest [8,9]. It is a widely used imaging technique that supports the surgeon i.a. to evaluate microvascular anastomosis and intra- and postoperative flap perfusion in real-time [10,11,12]. Further, preoperative imaging has gained popularity, especially in the planning of perforator flaps, as it delivers information about perforator course and caliber. Thereby, computed tomography angiography (CTA) has been widely considered the gold standard in cases of breast reconstructions with abdominal perforator flaps. Regarding donor site morbidity, in addition to fascia-preserving surgical techniques, the implantation of synthetic meshes to reinforce fascial closure to minimize the risk of postoperative abdominal hernia or bulge has been described over recent decades.

This study aimed to investigate the potential advantages of implementing technical innovations and utilizing a synthetic mesh for fascial donor site closure in both uni- and bilateral autologous breast reconstruction using abdominal tissue.

## 2. Materials and Methods

### 2.1. Patients

We performed a retrospective single-center chart review of 424 consecutive patients who underwent unilateral or bilateral abdominal-based autologous breast reconstruction with a DIEP or MS-TRAM flap between January 2004 and December 2019. The patients were informed in detail in advance about all treatment options, including alternative donor sites or—if possible—alloplastic reconstructive options. All included patients chose a breast reconstruction with abdominal tissue. Surgery reports and complete medical charts of the 424 patients were reviewed, and age at the time of surgery, body mass index (BMI), flap type, uni- or bilateral reconstruction, operation duration, and medical history including previous operations were recorded. Further, the application of technical devices including venous coupler systems, preoperative CTA, intraoperative ICG angiography, and the reconstruction of the donor site with fascial mesh repair were noted.

Complications were recorded and categorized into major complications (requiring surgical intervention) and minor complications (could be managed conservatively). We further distinguished between complications related to the donor and the recipient site. Major complications included complete or partial flap loss, the necessity of flap revision due to venous or arterial congestion, bleeding, umbilical necrosis, infection, seroma, and abdominal hernia/bulging. Conservatively addressed minor complications comprised wound dehiscence, wound infection, seroma, partial flap necrosis, and abdominal hernia/bulging. Thereby, every smallest wound healing disorder was scored and included. Follow-up was at least six months.

### 2.2. CTA

CTA was performed as described in detail previously [13]. Briefly summarized, CTA was conducted on a 128-slice CT scanner (SOMATOM AS+, Siemens Healthcare GmbH, Forchheim, Germany) with a defined scan range from the last thoracic vertebral body to the ischial tuberosity. Iodinated contrast agent (Iomeprol, Imeron 350, Bracco Imaging GmbH, Konstanz, Germany) was introduced intravenously through an antebrachial vein via power injector (Accutron CT-D, Medtron AG, Saarbrücken, Germany). For general image evaluation, multiplane reconstructions in the axial plane were created (slice thickness 5 mm, increment 5 mm, B30f smooth reconstruction kernel). With the processing of overlapping maximum intensity projection (MIP) images in the sagittal, axial, and coronal planes (slice thickness 10 mm, increment 5 mm, B30f), abdominal perforators could be visualized prior to surgery. The protocol for CTA processing was established in an interdisciplinary manner in collaboration with colleagues from the radiology department.

Before the use of CTA, a hand-held Doppler device was used for preoperative perforator detection.

### 2.3. Laser-Assisted ICG Fluorescence Angiography

Intraoperative laser-assisted ICG fluorescence angiography was conducted with the SPY Elite System (Novadaq Technologies Inc., Toronto, ON, Canada) as described earlier [12,14]. ICG (VERDYE Diagnostic Green GmbH, Aschheim-Dornach, Germany) was used in a concentration of 2.5 mg/mL. A 5 mL bolus was injected through a peripheral venous line followed by a flush of 10 mL of saline. The first ICG angiography perfusion analysis was performed after complete flap harvest and non- or hypoperfused areas of the flap were debrided. Second tissue perfusion was assessed at the end of the surgery at the recipient site after the insertion of the flap to verify overall sufficient flap perfusion.

### 2.4. Venous Coupler Anastomosis

For venous coupler anastomosis, a ring-pin coupler system from Synovis (St. Paul, MN, USA) was used. The correct coupler size was determined with a vessel-measuring gauge. After attachingthe donor vein, the recipient vein is connected to its coupling ring. The venous coupler anastomosis was performed as previously reported in detail by Jandali et al. [6].

### 2.5. Donor Site Closure

When possible, primary fascial closure was attempted following abdominal tissue harvest. When donor site closure was performed without mesh reinforcement, the fascia was sutured with multifilament U-technique sutures. Fascia doubling was performed with a monofilament running suture.

In the group of patients who received rectus sheath closure with mesh implantation, primary closure was reinforced by single-layered synthetic mesh implanted in continuous suture technique. A second suture was performed for fascial closure. When fascial defects were too large for direct fascial reapproximation, the rectus sheath was reconstructed by the interposition of a doubled synthetic mesh with continuous suture technique.

### 2.6. Statistics

Demographic data are presented as mean ± standard deviation (SD) and range. The normal distribution was analyzed by the Shapiro–Wilk test. Categorical outcomes were compared between groups using a Chi-square test or Fisher’s exact test. Continuous data were compared using the Kruskal–Wallis test, followed by Dunn’s test for post hoc analysis or ordinary one-way ANOVA. GraphPad Prism 9 (GraphPad Software, San Diego, CA, USA) was used for statistical analysis. A value of *p* ≤ 0.05 was considered significant.

## 3. Results

We performed a retrospective chart review of 424 patients that underwent uni- or bilateral abdominal-based free flap breast reconstruction between January 2004 and December 2019. Due to insufficient follow-up data, 28 patients (29 flaps) had to be excluded from further analysis. Table 1 provides a summary of the patient demographic characteristics. Of the 396 patients included, 51 patients (13%) received bilateral breast reconstruction. Ages ranged from 31 to 77 years (mean 52 years). For all procedures except prophylactic mastectomies, exclusively delayed secondary reconstructions were performed.

Over time, new technical tools have been developed and routinely used in the context of breast reconstruction with autologous tissue. Since February 2008, intraoperative venous coupler anastomoses replaced hand-sewn venous anastomoses within our patient collective. Additionally, preoperative CTA for perforator detection has been performed routinely since December 2009. Intraoperative ICG angiography has served as the intraoperative perfusion monitoring method since March 2014. The number of performed flaps according to the respective performed technical tools is summarized in Table 2. The impact of different technical innovations and the usage of graft materials for donor site closure were investigated in regards to the operation time and the occurrence of complications.

**Figure 1:**
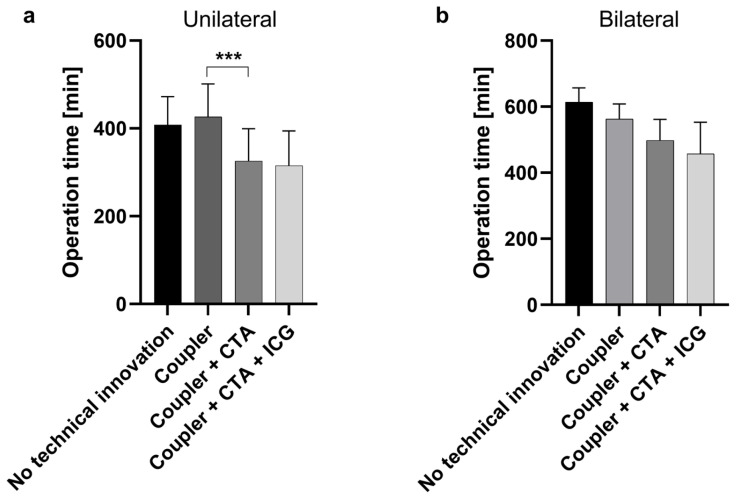
Operation duration in minutes for unilateral (**a**) and bilateral (**b**) breast reconstruction according to applied technical innovation. In unilateral reconstructions, operation times were significantly shorter after the implementation of preoperative computed tomography angiography (CTA) (Coupler + CTA vs. Coupler), ***: *p* < 0.0001. Groups were compared as follows: No technical innovations vs. Coupler, Coupler vs. Coupler + CTA, and Coupler + CTA vs. Coupler + CTA + ICG. (ICG: indocyanine green).

To determine whether the introduction of a new technical innovation was associated with a shorter operation time, the operation times were compared as follows: No technical innovations vs. Coupler, Coupler vs. Coupler + CTA, and Coupler + CTA vs. Coupler + CTA + ICG. Operation time in the coupler group was significantly longer than after the introduction of CTA (Figure 1). This significant time saving was seen only in unilateral flaps (*p* < 0.0001). The application of coupler anastomoses and ICG angiography has shown no influence on operation times.

### 3.1. Complications at the Recipient Site

Major complications at the recipient site occurred in 47 patients (11.9%). Within this group, three patients underwent bilateral breast reconstruction. Minor complications at the breast could be observed in 19 patients (4.8%). One of these patients received bilateral reconstruction. Table 3 summarizes major and minor complications at the recipient site by showing different types of complications and their distribution among patients.

**Figure 2:**
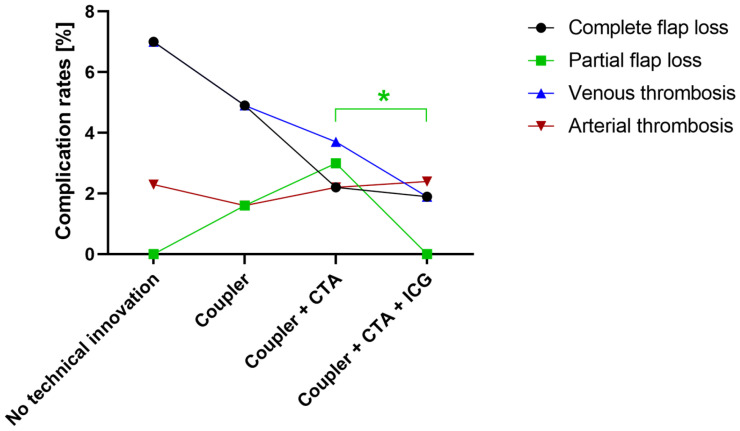
The distribution of complication rates of selected major complications according to applied technical tools. After the implementation of intraoperative indocyanine green (ICG) angiography, partial flap loss was significantly reduced. *: *p* < 0.05. (CTA: computed tomography angiography).

Altogether, venous thrombosis was observed in 15 patients. Within this group, five cases resulted in total flap loss. The application of venous coupler anastomoses did not result in an overall reduction in surgery duration compared to breast reconstruction without coupler anastomoses. Flap loss and venous congestion occurred in three cases (7%) with hand-sewn venous anastomoses and in three cases (5%) in which a coupling device was used as only technical tool (Figure 2). In total, since the introduction of venous coupler anastomoses, twelve cases of venous congestion (3%) have been noticed, of which three suffered complete flap loss.

A significant change in flap-specific complication rates of the recipient site with the introduction of perioperative CTA could not be observed.

The overall complication rate tended to be reduced by using intraoperative ICG angiography (*p* < 0.1). While ICG did not significantly decrease the occurrence of complete flap loss or venous or arterial thrombosis, it significantly reduced the rates of wound healing disorders (*p* = 0.02) and partial flap loss (*p* = 0.02) (Table 4, Figure 2).

### 3.2. Complications at the Donor Site

Complications at the donor site occurred more often compared to the recipient site. A total of 54 patients (13.6%) suffered from major complications; minor complications were observed in 129 cases (32.6%). The distribution of major and minor complications at the donor site is summarized in Table 5.

Eight patients undergoing unilateral breast reconstruction received a double-layer mesh implantation because the primary reconstruction of the fascia could not be performed due to the size of the fascial defect. In bilateral reconstructions (102 flaps), mesh repair was performed in 94 cases. Of these cases, anatomical reconstruction could be performed in 67 flaps (71%) and double-layer mesh repair was performed in 27 flaps (29%). A total of 73% of the patients underwent mesh repair with a synthetic titanium-coated polypropylene mesh (TiMESH^®^, pfm medical ag, Köln, Germany), 22% received a vicryl–prolene composite synthetic mesh (Vypro^®^, Ethicon, Johnson & Johnson, Norderstedt, Germany), and in 5% of the patients, further types of synthetic meshes were implanted (Prolene^®^ (Ethicon), Marlex^®^ (Ethicon)). No significant differences were found between the types of mesh and the occurrence of major or minor complications. The percentage of patients with previous abdominal surgery was approximately the same in the group without mesh implantation (67%) and the group with mesh implantation (65%) (*p* = 0.8).

The occurrence of major and minor complications and the use of mesh was analyzed separately for uni- and bilateral reconstructions. In unilateral reconstructions, conservatively-treated seroma developed significantly more often after mesh implantation (15.3% vs. 5.6%) (*p* = 0.03). Seroma requiring a return to the operating theatre was observed in 13 patients. Within this group, 10 patients had undergone mesh implantation (*p* = 0.8). Abdominal hernia/bulging requiring operative intervention was observed less frequently in patients with mesh implantation (2.9%) compared to those who received no mesh (12.7%) in unilateral reconstructions. However, no reliable conclusion can be drawn from this, as the type of flap plays a decisive role in the development of hernia/bulging and must be included in the evaluation (Table 6).

There was no difference concerning overall major donor site complications in unilateral (41 patients, 12%) and bilateral (7 patients, 14%) reconstructions (*p* = 0.7). However, comparing donor site complications in uni- and bilateral reconstructions with mesh repair, a significantly higher incidence of umbilical necrosis is noticeable with bilateral reconstructions (10.4% vs. 2.6% in unilateral reconstructions, *p* = 0.008). The mean BMI of patients suffering umbilical necrosis was 27.0 kg/m^2^, which corresponds to the average BMI of the total patient collective and to the average BMI of patients without umbilical necrosis. No significant difference between these groups was found concerning minor complication rates. Since the number of patients with bilateral reconstructions in our data is low (*n* = 51) and since different flap types were often combined in bilateral breast reconstruction, a further subdivision into complication rates per flap type for bilateral reconstruction is not possible in the current study. Therefore, our data do not allow us to conclude whether there is a correlation between umbilical necrosis and a specific type of flap. Further, due to the given small number of patients with bilateral reconstructions without mesh use (*n* = 3), no reliable conclusion can be drawn for this group.

**Figure 3:**
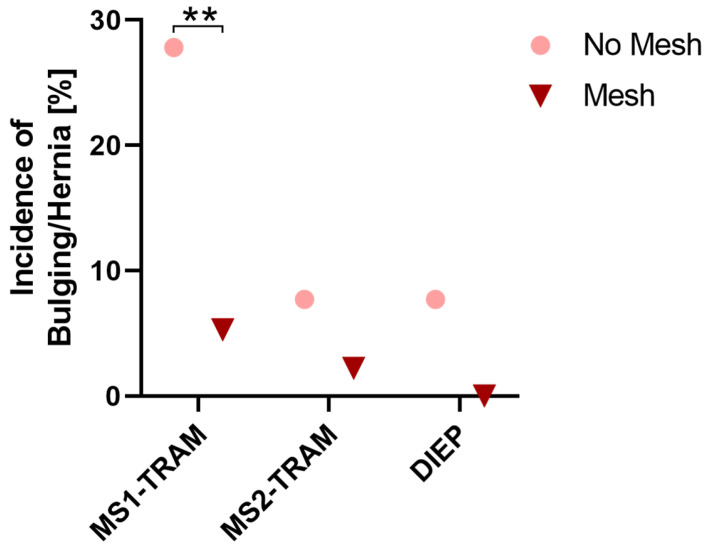
Incidence of bulging or hernia in unilateral reconstructions presented for MS1-TRAM, MS2-TRAM, and DIEP flaps with and without synthetic mesh repair. **: *p* < 0.01.

Regarding different flap types in unilateral reconstructions, the relative occurrence of major donor site complications was lowest in DIEP flaps, whereby no statistically significant difference was observed between different flap types (*p* = 0.1). Table 6 shows the incidence of major and minor complications according to flap type and mesh use. Relative major complication rates for MS1-TRAM, MS2-TRAM, and DIEP flaps were lower in cases with mesh repair. However, this was only statistically significant for MS1-TRAM flaps (*p* = 0.02). The incidence of the major complication of bulging/hernia was higher in patients without mesh repair in MS1-TRAM, MS2-TRAM, and DIEP flaps (Figure 3). This was statistically significant for MS1-TRAM flaps (*p* = 0.001). Bulging tended to decrease from MS1-TRAM via MS2-TRAM to DIEP flaps. The best results regarding the occurrence of bulging could be achieved in DIEP flaps with mesh repair (0% bulging). Similarly, no bulging/hernia occurred in MS0-TRAM cases. However, due to the small quantity, no reliable statement can be made about the incidence of bulging in MS0-TRAM flaps.

## 4. Discussion

Breast reconstruction with abdominal perforator flaps is a well-established and advanced surgical technique that has evolved to the gold standard for autologous breast reconstruction. Since its inception, further innovations and developments have been introduced for microsurgical flaps to optimize outcomes. The focus of this work was set on four techniques currently applied as the standard in our institution, including venous coupler anastomoses, preoperative perforator imaging with CTA, intraoperative ICG angiography, and mesh use for reinforcement fascial closure or fascial reconstruction, respectively. This study aims to address the potential benefits seen with the use of those methods on operative complications and surgical times.

After the introduction of the first metal coupling device in 1962 [15], microvascular anastomotic coupling devices were increasingly described in animal and clinical studies in the 1980s [16,17,18]. Their use has become widespread in recent decades, and they are now indispensable in a large proportion of microsurgical centers. Several studies showed a low incidence of intra- and postoperative venous thrombosis of less than 1% with the use of coupler devices and described the microvascular coupler as a reliable and fast method [6,19]. Further, operation time savings through the application of coupling devices have been described [20,21]. In the present study, the relative number of venous congestion and flap loss due to venous congestion decreased with the introduction of venous coupler devices. Due to the considerably lower number of cases of hand-sewn venous anastomoses and the generally rare occurrence of venous thromboses, no more reliable statement results from our data.

CTA was first described for imaging perforator anatomy in the planning of free abdominal-based breast reconstruction in 2006 [22,23]. Although CTA involves exposure to radiation and nephrotoxic contrast agents, it is, to some extent, still considered the gold standard in many centers due to its high reproducibility, reliability, and spatial resolution with a high level of sensitivity to perforator course and caliber [24,25]. Preoperative CTA assists in evaluating individual anatomical circumstances and, thus, identify suitable perforators. In addition to providing information about the number, caliber, and course of the perforators, CTA also provides information about the connection of the perforators to the superficial inferior epigastric vein [25,26]. This is crucial for the adequate venous drainage of the flap. There has been a number of studies describing a reduction in operative time and complication rates by introducing preoperative CTA scans for flap planning [27,28,29,30,31]. A previous study described a more pivotal advantage of preoperative CTA in the case of bilateral reconstructions [27]. Despite observing a reduction in operation times in uni- and bilateral reconstructions, we only found significant time savings in unilateral flaps. This might be due to the decisive assistance of preoperative CTA imaging in the selection of the appropriate donor site. This is only relevant in unilateral reconstructions so that a more targeted preparation can be performed on the more suitable side. Hence, preoperative perforator imaging such as CTA is a valuable tool that facilitates safe and rapid flap elevation for the surgeon.

In 2002, ICG angiography was initially described for intraoperative use in microvascular free flap reconstructions [32]. Over the years, the technique has continued to evolve and refine, becoming a valuable tool for monitoring blood flow in the vessels that supply the transplanted tissue during reconstructive procedures [8,33,34]. Thus, the surgeon is able to assess the perfusion of the flap in real time and make necessary patient-specific adjustments during the surgery. Given the variability in the perfusion pattern of each flap according to the location and number of the included perforators in abdominal-based autologous breast reconstruction, ICG angiography supports intraoperative decision making for individual flap tailoring [12]. In the present study, we observed a reduction in partial flap necrosis and wound healing disorders since the introduction of intraoperative ICG angiography application. This is in accordance with previous studies showing that ICG angiography in DIEP flap breast reconstructions significantly lowered the odds of fat necrosis and reoperations [35,36,37,38]. This innovation has significantly improved the success rates of microvascular free flap reconstructions by reducing the risk of partial flap necrosis. It is also worth mentioning that none of the patients experienced any undesirable side effects. Thus, ICG is a safe and reliable procedure that has become an indispensable part of modern autologous breast reconstruction. In addition to the intraoperative use of ICG, other studies have described the value of ICG in the detection of postoperative flap ischemia [39,40]. In the current study, postoperative flap monitoring was performed through clinical examination and Doppler ultrasound. Further technical innovations such as dynamic infrared thermography, hyperspectral imaging, or laser speckle contrast analysis have been proposed to support and refine flap monitoring in recent years [41]. Although clinical examination in combination with acoustic Doppler sonography is still considered the gold standard, novel technologies can objectify the assessment and support the examiner in decision making.

The success of reconstruction with autologous tissue depends not only on the results at the recipient site, but also on minimizing donor site morbidity. In abdominal-based autologous breast reconstruction, restoring the integrity of the abdominal wall is particularly important. To prevent the development of hernias or bulging, muscle-sparing and fascia-saving techniques have gained more importance. However, all these techniques involve disrupting the anterior rectus abdominis fascia, and as a result, the risk of developing bulging or hernias cannot be completely eliminated. Reinforcing fascial closure with meshes has been described as a promising strategy to avoid these complications [42]. Whereas a recent study proclaimed a safe performance of abdominal closure without graft materials, even in bilateral reconstruction [43], previous findings described bilateral flap harvest as a predictor of the increased incidence of postoperative abdominal bulge or hernia [44,45]. In the present study, we could not identify any significant differences regarding the development of bulging between unilateral and bilateral reconstruction with mesh repair, suggesting that with mesh implantation, bilateral reconstruction can be performed safely. Further, earlier findings revealed a seven to eight percent rate of hernia or bulge formation that necessitated reoperation [44,46]. Our data showed that hernia or bulging occurred in less than three percent of uni- and bilateral reconstructions using mesh repair. Additionally, comparing unilateral reconstructions with the implantation of meshes compared to those without meshes, we observed a significantly lower incidence of bulging or hernia. Considering the occurrence of bulging and hernias in the different flap types, it is noticeable that this complication occurred mainly in MS1-TRAM flaps without mesh. In unilateral reconstructions, the incidence of bulging and hernia in MS1-TRAM flaps was significantly reduced by mesh repair. For MS2-TRAM and DIEP flaps, bulging/hernia appeared less frequently. Hernia/bulge rates in those flap types were further reduced by mesh implantation, although the difference between reconstructions with mesh implantation was not significant. Thus, the importance of mesh implantation decreases with muscle-sparing techniques, which is also consistent with previous data [47]. However, it is noteworthy that among the 93 patients who received a DIEP flap with mesh repair for unilateral reconstruction, none developed bulging or hernia. This result is lower compared to a previous study that reported an incidence of bulge or hernia of 2.1 percent in patients undergoing a DIEP flap reconstruction without mesh implantation [45]. In the event of a post-operative hernia, an interdisciplinary approach involving general surgeons should be considered. Data from unilateral reconstructions showed that the flap type has an influence on the development of bulging and hernias. Since the number of bilateral reconstructions in our data is low and since different flap types were often combined in bilateral breast reconstruction, a further subdivision into complication rates per flap type for bilateral reconstruction is not possible in the current study. Therefore, no reliable conclusion concerning bulging or hernia could be drawn for bilateral reconstructions.

One disadvantage of mesh implantation revealed by our study is the increased occurrence of seroma in unilateral reconstructions with mesh repair. For bilateral reconstructions, seroma formation could not clearly be assigned retrospectively to one side. Further, in some patients with bilateral reconstruction, mesh repair was performed only on one side. Therefore, no conclusions can be drawn about mesh implantation and seroma formation in bilateral reconstructions. Seroma formation is a known donor site complication after abdominal-based autologous breast reconstruction. In the majority of cases, seroma can be treated successfully with percutaneous aspiration, or it resolves spontaneously. Nevertheless, seroma may have deleterious consequences with a substantial risk of wound-related complications, including infection or wound dehiscence. Visceral surgeons have already described that seroma and total wound complications occur more frequently after ventral hernia repair with mesh compared to non-mesh repair [48]. Within our patient collective, we observed a higher incidence of seroma that could be managed conservatively after mesh implantation. However, there was no difference in the occurrence of seroma that could not be managed with conservative therapy between mesh and non-mesh repair. According to the authors, the benefit of reduced bulging from mesh implantation outweighs the risk of increased seroma. Non-synthetic mesh alternatives such as acellular dermal matrices (ADM) might also be considered for reinforcing abdominal donor sites following flap harvest in selected cases [49,50,51,52]. ADM are attributed to a lower infection rate and better incorporation into the surrounding tissues compared to synthetic meshes [53]. A meta-analysis described a seroma rate of approximately 12% when applying ADM in the context of abdominal wall reconstruction [54]. Results of ADM abdominal wall reinforcement regarding bulging or hernias were variable [50,54]. Further promising materials ameliorating the reinforcement of the abdominal wall might be explored by scientific advances in tissue engineering approaches in the future [55].

Another donor site complication about which patients should be informed is the risk of umbilical necrosis. Recent findings described that radiographic measurements of umbilical stalk height/abdominal wall thickness ratio could reliably predict the appearance of umbilical complications [56]. Further, Ricci et al. showed a correlation with flap weight, increased number of perforators per flap, higher body mass index, older age, and smoking. In our patient collective, we found a significantly higher rate of umbilical necrosis in bilateral than in unilateral reconstructions. This is in accordance with previous observations of an association between umbilical necrosis and bilateral reconstruction [57]. In bilateral dissection, the median umbilical ligament and the ligamentum teres hepaticum are potentially the only sources of perfusion to the umbilicus, leaving the umbilicus more vulnerable [58]. Patients should be aware of that risk, especially if they have had previous operations that might have compromised perfusion via the above-mentioned structures. In certain cases, umbilectomy might be even indicated to minimize donor site complications [59,60]. Overall, donor site morbidities might occur in uni- and bilateral reconstructions and in all types of flaps; so, having a successful breast reconstruction might imply having to bear complications in the donor area in some cases. Patients need to be well informed about the potential risk of these donor site morbidities during consultation by the surgeon.

There are limitations to this study, which are related in particular to the retrospective design. The group sizes are variable, and in some cases, the groups include only small numbers of patients, so that a reliable statement is not possible for all investigated aspects. Some complications occurred very rarely in both comparable groups, so that the risk of confounding variables cannot be excluded in these cases. Another limitation of this study is that due to the retrospective analysis over a long period, a potential confounding factor due to gradual progression over time and the surgeons’ individual learning curves cannot be completely ruled out. However, we report data from an academic teaching institution, and the data are based on different surgeons. We performed a single-center study and there are standardized regulations during the procedure and in postoperative care. All participating surgeons are very experienced microsurgeons. Nevertheless, since different surgeons at our clinic have always had a possible learning curve over the years, the timing of the introduction of newer techniques is rather independent of the individual learning curves. Additionally, information regarding fascial defect size and tension of repair at the donor site could not be retraced, since measuring suture tension in every patient is not a routine procedure [61]. Further, postoperative outcomes might be influenced by intraoperative perfusion management [62]. The current retrospective study lacks data regarding intraoperative blood pressure or volume management, thereby presenting another limitation of the study.

There exist various factors that may affect the outcomes of breast reconstruction, alongside the incorporation of contemporary techniques and the decision-making process surrounding their utilization. This necessitates meticulous analysis and collaboration across multiple disciplines. As for future directions, patients undergoing breast reconstruction might benefit from new promising technologies that have recently been described. For preoperative perforator mapping, a new three-dimensional visualization technique, cinematic rendering, was described as more photorealistic with improved detail images compared with other 3D reconstruction techniques [63]. It can assist less experienced surgeons in particular in a faster and better understanding of the individual anatomy [13,64]. Others have compared the value of 3D-printed models from CT perforator scans and their virtual renderings to superimpose relevant imaging on a surgeon’s native field of view for teaching purposes and to alleviate the dissection of anatomically variable structures [65]. Further, the assessment of magnetic resonance imaging or CT data might be carried out on the patient’s body itself and not only on the computer. Projection mapping was described as promising tool to fill the gap between preoperative planning on the computer and surgery on the patient [66]. Thus, instead of approximate measurement, the surgeon would be able to see the individual perforator anatomy directly on the abdominal wall of the patient. For optimizing intraoperative procedure, robotic technology aims to increase surgical precision, therefore minimizing complications [67,68,69]. The clinical significance of these innovations must be demonstrated in the following years.

## 5. Conclusions

Patients undergoing abdominal-based autologous breast reconstruction can benefit from the application of technical innovations before and during the surgical procedure to optimize outcomes. ICG angiography was associated with reduced rates of partial flap loss and wound healing disorders. Abdominal bulging or hernia occurred less frequently after synthetic mesh repair in MS1-TRAM flaps in unilateral reconstructions. However, one disadvantage of synthetic mesh implantation is its association with a higher incidence of conservatively treated seroma. Operation time was significantly shorter in unilateral reconstructions after the implementation of CTA.

## Figures and Tables

**Table 1:** Demographic data and distribution of flaps.

Number of patients	396
Age at operation date (mean ± SD; range) [years]	52 ± 9; 30–77
BMI (mean ± SD; range) [kg/m^2^]	27 ± 4; 17–45
Number of flaps DIEP flaps MS2-TRAM flaps MS1-TRAM flaps MS0-TRAM flaps	447 140 (31%) 109 (25%) 192 (43%) 6 (1%)

**Table 2:** Distribution of flaps according to technical innovations.

	No. of Flaps	Age (Mean ± SD)	
No technical innovation	44 (9.8%)	52.1 ± 9.4	*p* = 0.98
Coupler anastomosis	61 (13.7%)	52.3 ± 8.2	
Coupler anastomosis + CTA	134 (30%)	51.8 ± 9.2	
Coupler anastomosis + CTA + ICG angiography	208 (46.5%)	52.0 ± 8.4	

CTA: computed tomography angiography, ICG: indocyanine green.

**Table 3:** Major and minor complications observed at the recipient site.

Type of Major Complication	No. of Cases (%)	Type of Minor Complication	No. of Cases (%)
Total flap loss	13 (3.3)	Wound dehiscence	12 (3.0)
Partial flap loss	5 (1.3)	Seroma	4 (1.0)
Venous thrombosis	15 (3.8)	Infection	2 (0.5)
Arterial thrombosis	10 (2.5)	Partial flap necrosis	1 (0.3)
Hematoma/bleeding	15 (3.8)		
Infection	9 (2.3)		
Seroma	1 (0.3)		

**Table 4:** Number of cases (%) of recipient site complications according to applied technical innovations.

Type of Major Complication	Total Flap Loss	Partial Flap Loss	Venous Thrombosis	Arterial Thrombosis	Hematoma/ Bleeding	Infection	Seroma
No. technical innovation	3 (7.0%)	0 (0%)	3 (7.0%)	1 (2.3%)	2 (4.7%)	1 (2.3%)	0 (0%)
Coupler anastomosis	3 (4.9%) *p* = 0.69	1 (1.6%) *p* > 0.99	3 (4.9%) *p* = 0.69	1 (1.6%) *p* > 0.99	3 (4.9%) *p* > 0.99	4 (6.6%) *p* = 0.40	1 (1.6%) *p* > 0.99
Coupler anastomosis + CTA	3 (2.2%) *p* = 0.34	4 (3.0%) *p* > 0.99	5 (3.7%) *p* = 0.70	3 (2.2%) *p* > 0.99	6 (4.5%) *p* > 0.99	2 (1.5%) *p* = 0.08	0 (0%) *p* = 0.31
Coupler anastomosis + CTA + ICG angiography	4 (1.9%) *p* > 0.99	0 (0%) * *p* = 0.02	4 (1.9%) *p* = 0.32	5 (2.4%) *p* > 0.99	4 (1.9%) *p* = 0.20	2 (1.0%) *p* = 0.65	0 (0%) *p* > 0.99

*p* values are from the Fisher’s test, *: *p* < 0.05 Groups were compared as follows: No technical innovations vs. Coupler, Coupler vs. Coupler + CTA, and Coupler + CTA vs. Coupler + CTA + ICG.

**Table 5:** Major and minor complications observed at the donor site.

Type of Major Complication	No. of Cases (%)	Type of Minor Complication	No. of Cases (%)
Abdominal hernia/bulging	21 (5.3)	Abdominal hernia/bulging	18 (4.5)
Seroma	13 (3.3)	Seroma	56 (14.1)
Infection	9 (2.3)	Infection	6 (1.5)
Hematoma/bleeding	4 (1.0)	Wound dehiscence	61 (15.4)
Umbilical necrosis	19 (4.8)		

**Table 6:** Major and minor donor site complications in unilateral abdominal-based breast reconstruction according to different flap types and synthetic mesh repair. *: *p* < 0.05, **: *p* < 0.001, n.s.: not significant.

	No.	Major Donor Site Complications	*p* Value	Minor Donor Site Complications	*p* Value
MS0-TRAM - Mesh - No mesh	6 5 1	0 (0%) 0 (0%) 0 (0%)	--	0 (0%) 0 (0%) 0 (0%)	--
MS1-TRAM - Mesh - No mesh	149 131 18	28 (19%) 21 (16%) 7 (39%)	* *p* = 0.02	49 (33%) 48 (37%) 1 (6%)	** *p* < 0.001
MS2-TRAM - Mesh - No mesh	84 45 39	9 (11%) 4 (9%) 5 (13%)	n.s.	21 (25%) 13 (29%) 8 (21%)	n.s.
DIEP - Mesh - No mesh	106 93 13	4 (4%) 3 (3%) 1 (8%)	n.s.	28 (26%) 25 (27%) 3 (23%)	n.s.

## Data Availability

The datasets generated and/or analyzed during the current study are available from the corresponding author on reasonable request.
